# Invasion Risk of *Cosmos bipinnatus* in Xizang: A MaxEnt‐Based Assessment of Current Distribution and Future Risk Scenarios

**DOI:** 10.1002/ece3.72728

**Published:** 2025-12-17

**Authors:** Xin Tan, Zhefei Zeng, Wei Li, Jifeng Zhang, Norzin Tso, Ngawang Norbu, Mei Zhang, La Qiong, Junwei Wang

**Affiliations:** ^1^ Key Laboratory of Biodiversity and Environment on the Qinghai‐Tibetan Plateau, School of Ecology and Environment, Ministry of Education Xizang University Lhasa China; ^2^ Xizang Yani Wetland Ecosystem Positioning Observation Research Station Nyingchi China

**Keywords:** climate change, *Cosmos bipinnatus*, environmental variable, invasive plant, MaxEnt, spatial distribution

## Abstract

Under the backdrop of globalization, biological invasions driven by human activities have become a major threat to global biodiversity. As a crucial ecological security barrier in southwestern China, Xizang possesses rich biodiversity and fragile ecosystems. *Cosmos bipinnatus* , a widely cultivated ornamental invasive plant in Xizang, is spreading very fast and poses a potential threat to Xizang. Clarifying its distribution patterns and response mechanisms under climate change is critical for safeguarding regional ecological security and implementing effective invasion control measures. Through field investigations and computational modeling using optimized Maximum Entropy (MaxEnt) models with parameter tuning, ArcGIS 10.4, and R programming, we predicted current and future (2050 and 2090) potential invasion risk zones under two greenhouse gas emission scenarios (SSP1‐2.6 and SSP5‐8.5). MaxEnt results show: (1) The model's AUC of 0.983 indicates high accuracy. The dominant factors affecting *Cosmos bipinnatus* distribution are the average temperature of the coldest quarter, Human Impact Index, and upper soil pH. (2) Under future climates, the potential risk area of *Cosmos bipinnatus* will likely expand northwest. Under SSP1‐2.6, the risk area within Shannan City will shift. Under SSP5‐8.5, *Cosmos bipinnatus* is more sensitive to climate change, with faster expansion and longer–distance shifts, moving from Gacha County to Namling County. This research provides essential ecological evidence and practical reference for comprehensive prevention and dynamic monitoring of *C. bipinnatus* invasions in Xizang.

## Graphical abstract

Bio11 was the most important environmental variable for *C. bipinnatus* 's future distribution. HII would be an essential variable during the expansion of *C. bipinnatus* . High greenhouse gas emission trajectories significantly facilitate *C. bipinnatus* invasion across Xizang Autonomous Region under climate warming Projection of *C. bipinnatus* . Under contrasting SSP scenarios demonstrate convergent northwestward centroid migration toward northern plateau regions.

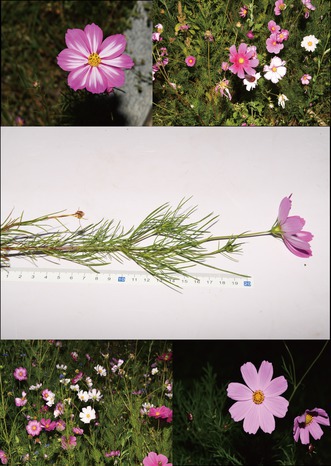

## 1 Introduction

Invasive alien species (IAS) refer to organisms that expand into new ecosystems through anthropogenic or natural means, subsequently undermining local biodiversity and disrupting ecological equilibrium (Zhang et al. 2021). In recent years, under the context of globalization, human activities such as economic trade have created opportunities for successful biological invasions. Biological invasions have emerged as a leading cause of biodiversity loss and global economic damage, evolving into a global ecological issue threatening natural ecosystems (Diagne et al. 2021). Therefore, identifying the current distribution and potential expansion areas of invasive plants is of great practical significance for early warning and management strategies.

Global climate change is reshaping the structure of ecosystems and distribution patterns of species at an unprecedented rate, exacerbating the risk of the spread of invasive alien species (IAS) (Wang et al. 2019; Clements and Jones 2021). Temperature changes, altered precipitation patterns, and the frequent occurrence of extreme weather events because of climate change provide favorable conditions for the spread of IAS, which typically have high reproductive capacity, high ecological plasticity, and strong resource competitiveness (Ahmad et al. 2023), characteristics that enable them to rapidly adapt to new environments in the face of climate change and establish dominant populations in a short period of time, thus posing a threat to local ecosystems and biodiversity (Zhang et al. 2021).

Xizang, a core component of the Qinghai‐Xizang Plateau, constitutes a vital ecological security barrier for China and ranks among the world's most biologically diverse highland regions (Wang, Zeng, et al. 2024; Chu et al. 2024). The intensification of human activities in the Xizang region has created favorable conditions for the spread and colonization of invasive plant species. Against the backdrop of global climate change, the region is undergoing significant ecological transformations, including a trend toward a warmer and wetter climate (He et al. 2023; Li et al. 2010), an increase in the frequency of extreme climate events, and a rise in greenhouse gas emissions. These changes are not only reshaping the local ecological environment but also presenting new challenges to species adaptability. Invasive plant species in the Xizang region, particularly (Lu et al. 2013), armed with their robust ecological adaptability, are swiftly colonizing and spreading under dynamically changing climatic conditions. This process is inflicting further damage on the fragile ecosystem of Xizang, exacerbating ecosystem instability and biodiversity loss. Research findings indicate that as many as 112 invasive plant species have been detected in Xizang. These invasive species are disrupting the original ecological pattern of the Tibetan Plateau and posing serious ecological threats to the region's unique highland environment (Chu et al. 2024).

*Cosmos bipinnatus* (Asteraceae), an annual herb native to Mexico, is commonly known as garden cosmos or Mexican aster (Olajuyigbe and Ashafa 2014). Characterized by semi‐hardiness and edaphic tolerance (Olajuyigbe and Ashafa 2014), this species has become naturalized across urban areas of the Xizang Autonomous Region. Current research on *C. bipinnatus* predominantly focuses on plant morphology (Paniagua‐Ibáñez et al. 2015), restoration ecology (Jadaun and Pandey 2024; Liu et al. 2017), invasion ecology (Datiles 2022), genomic architecture (Wanget, Xu, et al. 2024), and metabolomic/transcriptomic profiles (Wang et al. 2025). Nevertheless, critical knowledge gaps persist regarding its spatial distribution patterns and range expansion dynamics within Xizang's unique biogeographic context. Systematic investigation of its current distribution, invasion risk assessment, and predictive range modeling at the Xizang scale, therefore, carries significant ecological and management implications.

The Maximum Entropy (MaxEnt) model, a widely utilized tool for modeling species distribution patterns, has been extensively applied to assess climate change impacts on species distributions (Zhao et al. 2022; Sorbe et al. 2023), delineate conservation ranges for endangered species (Gao et al. 2022), and predict invasion hotspots of alien plants (Liu et al. 2023). Although existing studies have primarily focused on global (Zhang et al. 2023; Li et al. 2022), national (Zhang et al. 2020), or macro‐regional scales such as the Qinghai‐Xizang Plateau (Chai et al. 2025), research targeting the Xizang Autonomous Region remains scarce (Wang, Zeng, et al. 2024). This study addresses this gap by combining MaxEnt with ArcGIS to map the current distribution of the invasive plant *Cosmos bipinnatus* in Xizang, simulate its potential range expansion under SSP1‐2.6 and SSP5‐8.5 scenarios for 2050 and 2090, and identify key environmental drivers, including climatic factors, soil properties, elevation, and the Human Impact Index. Through the integration of multisource environmental variables, we delineate invasion risk zones across Xizang's heterogeneous landscapes, advancing both theoretical frameworks for high‐altitude invasion ecology and actionable strategies for adaptive management in Xizang and analogous Himalayan ecosystems.

## 2 Materials and Methods

### 2.1 Sources of Data on the Distribution of *C. bipinnatus*

Through systematic field investigations conducted during summer growing seasons (October 2022 to August 2024), we obtained georeferenced occurrence records of *C. bipinnatus* using standardized line‐transect surveys. The sampling framework prioritized prefecture‐level municipalities with intensive anthropogenic disturbances across the Xizang Autonomous Region, focusing on two predominant invasion‐prone habitats: (1) urban demolition sites within county‐level settlements and (2) road verges along primary transportation corridors.

**FIGURE 1:**
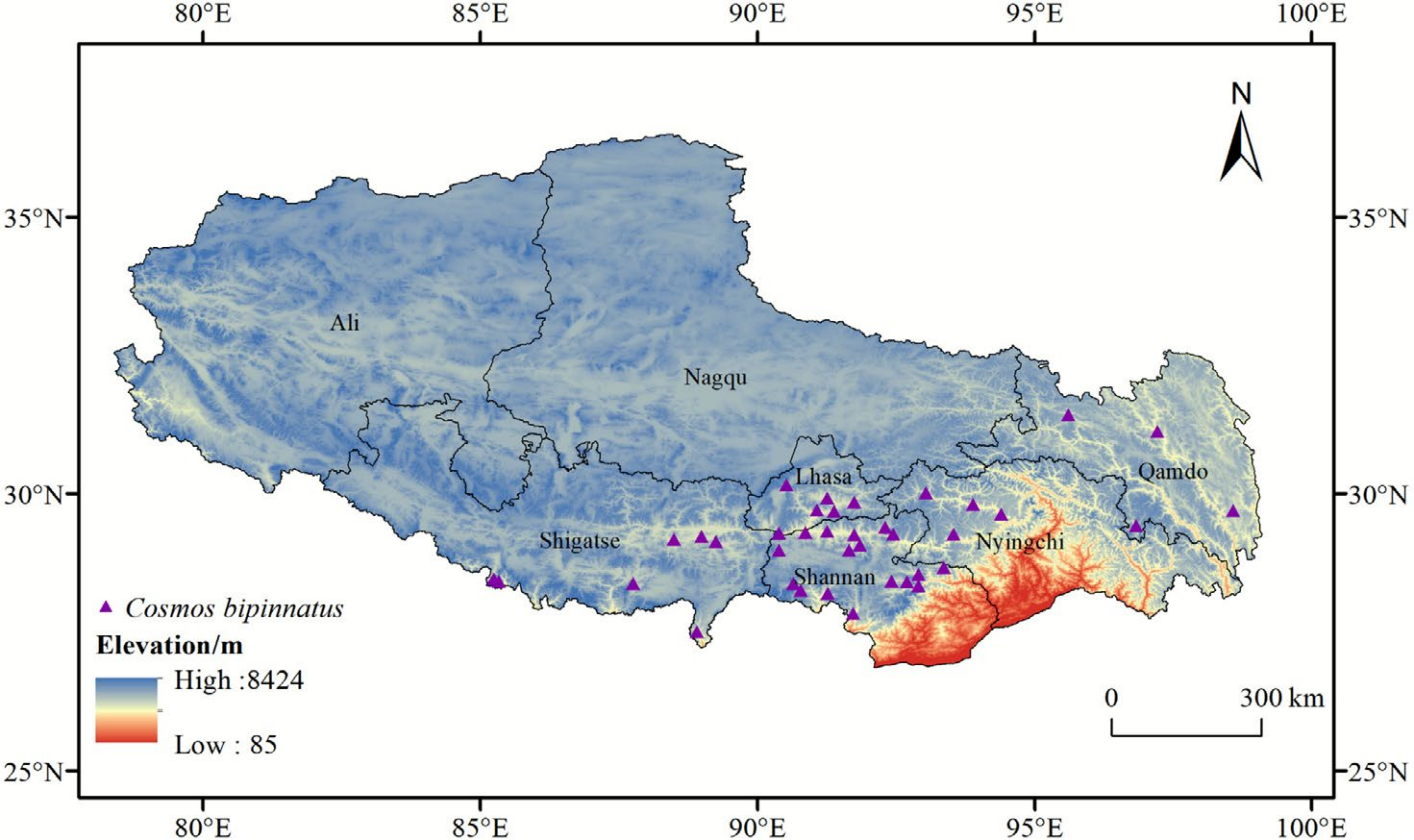
Location map of the study area in Xizang showing *C. bipinnatus* occurrence points.

The survey area encompassed the following regions (Figure 1), and a total of 68 distribution points of *C. bipinnatus* were documented during the field survey. The ArcGIS 10.4 software tool “Buffer Zone” was utilized to establish a buffer zone with a radius of 10 km. The distribution points that overlapped the buffer zone were excluded to ensure that the distance between two distribution points was greater than 10 km. A total of 38 field distribution points of *C. bipinnatus* were collected to minimize the effect of spatial autocorrelation. The 38 *C. bipinnatus* field distribution data were finally converted to the Comma Separated Values (.csv) format to prepare for the Maxent modeling and simulation prediction.

### 2.2 Pre‐Processing and Screening of Environmental Variables

The analysis incorporated 26 environmental variables comprising four categories: 19 bioclimatic factors, 3 soil characteristics, 3 terrain attributes (elevation, slope, aspect), and the Human Impact Index (Table 1). These parameters were integrated into the species distribution model to conduct habitat suitability projections, following established invasive species modeling methodologies (González‐Moreno et al. 2014).

**TABLE 1:** List of 26 environmental variables used in this study.

Data type	Variables	Descriptions	Units
Climate factor	Bio1	Mean annual temperature	°C
	Bio2	Mean monthly temperature range	°C
	Bio3	Isothermality	/
	Bio4	Temperature seasonality	/
	Bio5	Max temperature of the warmest month	°C
	Bio6	Min temperature of the coldest month	°C
	Bio7	Temperature annual range	°C
	Bio8	Mean temperature of the wettest quarter	°C
	Bio9	Mean temperature of the driest quarter	°C
	Bio10	Mean temperature of the warmest quarter	°C
	Bio11	Mean temperature of the coldest quarter	°C
	Bio12	Annual precipitation	mm
	Bio13	Precipitation of the wettest month	mm
	Bio14	Precipitation of the driest month	mm
	Bio15	Precipitation seasonality	mm
	Bio16	Precipitation of the wettest quarter	mm
	Bio17	Precipitation of the driest quarter	mm
	Bio18	Precipitation of the warmest quarter	mm
	Bio19	Precipitation of the coldest quarter	mm
Landform factor	Elev	Elevation	m
	Slope	Slope	%
	Aspect	Aspect	°
Soil factor	AWC	Available water storage capacity in mm/m of the soil unit	%
	T_pH_H_2_O	Soil reaction of the upper soil	log(H^+^)
	S_pH_H_2_O	Soil reaction of the lower soil	log(H^+^)
Human activity factors	HII	Human Impact Index, the Human Footprint	/

The environmental variables comprising 19 bioclimatic variables and 3 topographic parameters were obtained from the WorldClim database (https://worldclim.org/; accessed April 15, 2025) at 30‐arcsecond resolution (1 km^2^). Baseline climate layers represent the 1970–2000 climatological normal derived from observational records. These raster datasets were processed through georeferencing, clipping, and masking procedures against the official administrative boundaries of Xizang Autonomous Region using ArcGIS 10.4 (https://www.arcgis.com/, accessed 15 April 2025) and were subsequently converted into ASCII grid format (ASC) for ecological modeling applications.

Future bioclimatic projections encompassing mid‐century (2050) and end‐of‐century (2090) timelines were derived from the BCC‐CSM2‐MR global climate model within the CMIP6 framework under SSP1‐2.6 and SSP5‐8.5 scenarios, accessed through WorldClim version 2.1 (https://worldclim.org/; accessed 15 April 2025). These 30‐arcsecond (1 km^2^) resolution datasets comprised four scenario‐temporal combinations: 2050‐SSP1‐2.6, 2050‐SSP5‐8.5, 2090‐SSP1‐2.6, and 2090‐SSP5‐8.5.

Edaphic parameters were supplemented by the Harmonized World Soil Database v1.2 from the Food and Agriculture Organization (https://www.fao.org/soils‐portal/; accessed 15 April 2025). Anthropogenic pressure was quantified using the 2020 Human Influence Index (HIIv2) from the Wildlife Conservation Society (https://wcshumanfootprint.org/; dataset accessed via https://storage.googleapis.com/hii‐export/2020‐01‐01/hii_2020‐01‐01.tif; accessed 15 April 2025).

All environmental layers were spatially homogenized through reprojection and masking operations aligned with Xizang Autonomous Region's administrative boundaries. Raster resampling techniques maintained 30‐arcsecond grid consistency prior to conversion into a standardized ASCII format for ecological niche modeling.

**FIGURE 2:**
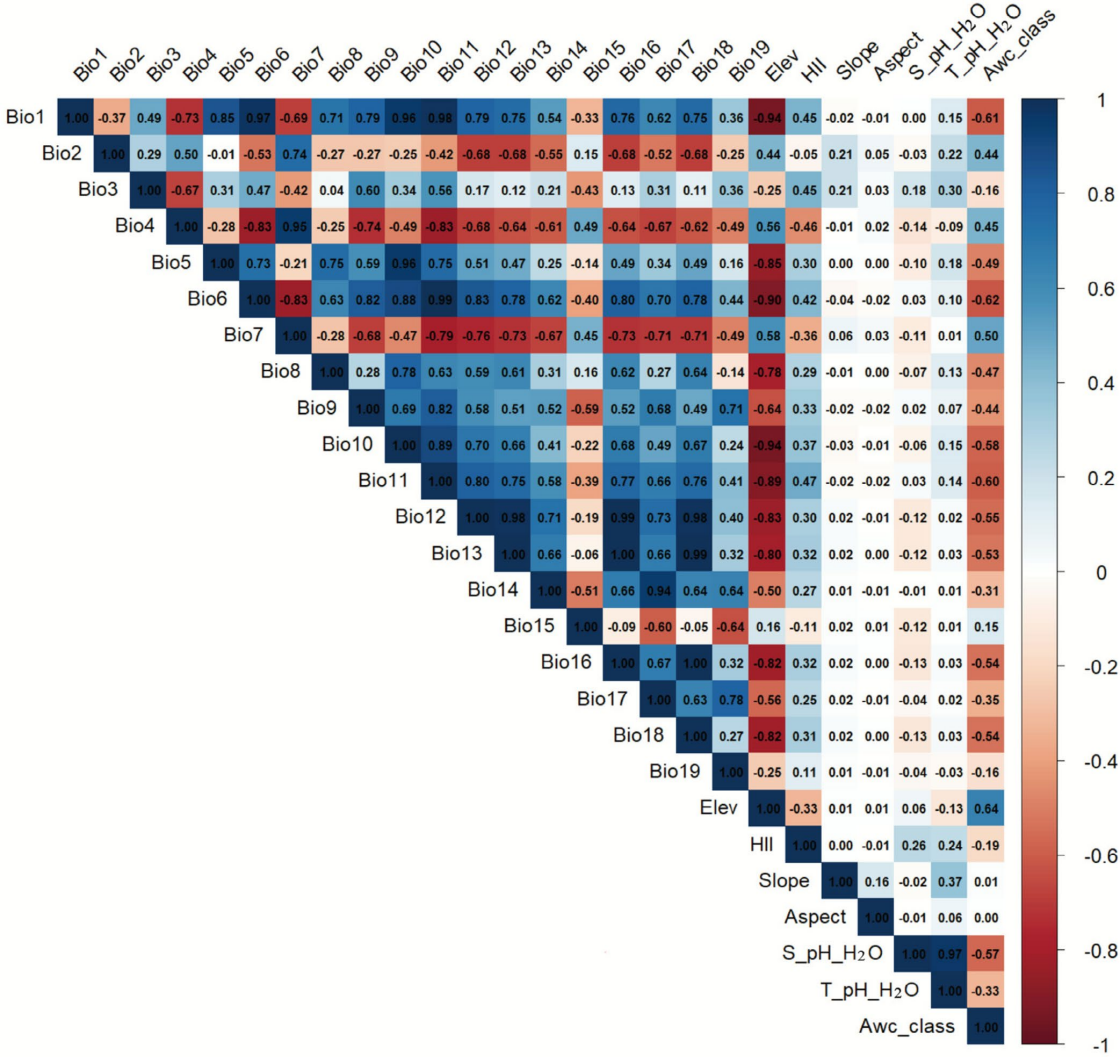
Correlation analysis of 26 environmental variables.

In order to avoid overfitting caused by multicollinearity and autocorrelation existing in environmental variables during the modeling process, we performed correlation analysis by EMNtools (https://github.com/danlwarren/ENMTools, accessed 15 April 2025) and primary pre‐modeling by Maxent (Li et al. 2023) on 26 environmental variables (Figure 2), retaining the environmental variables with |*r*| < 0.8 for secondary pre‐modeling (Graham 2003); for environmental variables with |*r*| > 0.8, according to the ranking of contribution rate and importance value in the pre‐modeling, environmental variables with zero contribution rate and lower importance value were screened out, and the environmental variables with higher contribution rate were considered (Simberloff et al. 2013). Considering the effects of soil physicochemical factors on plant invasion, and the fact that, in addition to climatic environmental variables, the impact of human activities is an important factor driving biological invasion (Chapman et al. 2020), and combining with the relevant studies of the previous researchers, a total of 8 environmental variables, including three climatic factors, two topographic factors, two soil factors, and one human impact index, were identified to participate in the ecological invasion of *C. bipinnatus* in the maximum entropy model prediction of ecological invasion. In order to ensure the comparability of the model in time series and the implementability of the runs, it was assumed that the 2 soil factors, 2 terrain factors, and the human impact index would remain constant in the next two time periods (2050, 2090).

### 2.3 Optimization of the Maxent Model

The predictive outcomes of Maxent are closely associated with its feature combination (FC) parameters and regulation multiplier (RM). It has been observed that default settings frequently result in a reduction of predictive accuracy (Hallgren et al. 2019). This study employed the “kuenm” package of R 4.2.3 (https://www.R‐project.org/, accessed 15 April 2025) to optimize Maxent (Zhang et al. 2022). The optimal parameters were determined to be Q and P (quadratic and product features) for feature combinations and 1.2 for the regulation multiplier.

### 2.4 Construction and Evaluation of the Maxent Model

Maxent version 3.4.4 (https://biodiversityinformatics.amnh.org/open_source/maxent/, accessed 15 April 2025) was implemented for ecological niche modeling of *C. bipinnatus* because of its demonstrated efficacy in species distribution prediction. Model construction incorporated 38 georeferenced occurrence records (.csv) and 8 optimized environmental variables. Parameter configuration included quadratic (Q) and product (P) feature classes with a regularization multiplier set to 1.2.

Model performance was evaluated through area under the receiver operating characteristic curve (AUC) analysis, following established validation protocols (Hosni et al. 2020). The AUC metric demonstrated: 0.5–0.6: Model performance equivalent to random prediction; 0.7–0.8: Moderate predictive accuracy; 0.8–0.9: Substantial discrimination capacity; 0.9–1.0: Excellent predictive capacity (Swets 1988).

The modeling framework incorporated 38 georeferenced occurrence records of *C. bipinnatus* (.csv) with 8 environmental variables in Maxent 3.4.4. Parameter configurations were set as follows: quadratic (Q) and product (P) feature classes with a regularization multiplier of 1.2; response curve visualization and jackknife analysis were enabled. In basic settings, random seed initialization was activated using a subsampling validation approach (75% training data vs. 25% testing data), with maximum background points constrained to 10,000. Advanced configurations included plot data output generation and iterative convergence thresholds set at 500 maximum iterations. The modeling protocol was executed through 10 independent model replicates to assess prediction stability. The predictive outputs of *C. bipinnatus* habitat suitability (ASCII format) for contemporary and two future periods in Xizang Autonomous Region were processed through reclassification procedures in ArcGIS 10.4 (ESRI). Using the Maximum Test Sensitivity Plus Specificity (MTSPS) threshold value of 0.0951 derived from Maxent model calibration (Aidoo et al. 2022), raster layers were dichotomized into potential risk areas (*P* ≥ MTSPS) and non‐risk zones (*P* < MTSPS). Spatial distribution centroid dynamics were quantified through SDMToolbox version 2.4 (http://sdmtoolbox.org, accessed 15 April 2025) integrated with ArcGIS geoprocessing framework. Centroid coordinate shifts across climate scenarios were analyzed to assess climate‐driven distribution pattern alterations.

## 3 Result

### 3.1 Analysis of the Assessment of the Contribution of Environmental Variables and Maxent's Maximum Entropy Model Accuracy Test

**FIGURE 3:**
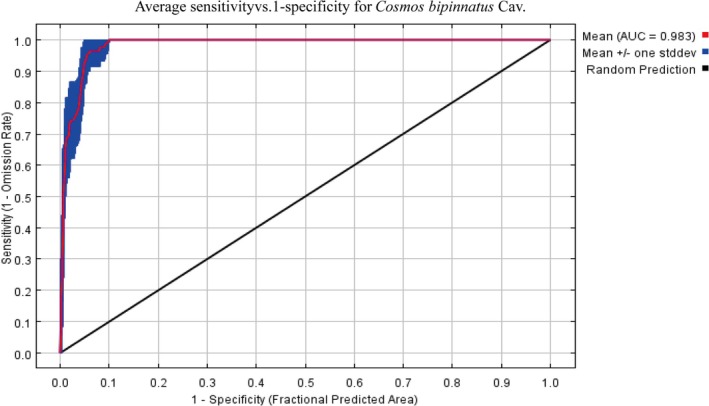
Area under the Receiver Operating Characteristic (ROC) curve for the training data, averaged over the replicate runs. The specificity was based on the predicted area. The average training AUC for the replicate runs was 0.983.

The resultant model demonstrated exceptional discriminatory power, yielding a mean area under the receiver operating characteristic curve (AUC) of 0.983 (Figure 3). This AUC value significantly exceeded random prediction levels (AUC = 0.5), confirming strong concordance between model projections and field‐validated occurrence records. Spatial predictions of *C. bipinnatus* invasion risk across Xizang showed robust consistency with observed colonization patterns, particularly in human‐modified habitats along transportation corridors.

Model diagnostics with jackknife permutation analysis revealed distinct contribution hierarchies among the 8 environmental variables (Table 2). The maximum entropy framework identified 3 dominant drivers: mean temperature of the coldest quarter (Bio11, 48.5%), HII (22.5%), and upper soil pH (T_pH_H2O, 21.5%), collectively explaining 92.5% of the model's predictive power. The three predominant variables cumulatively accounted for 92.5% of model explanatory power, establishing their role as principal determinants of *C. bipinnatus* distribution patterns in contemporary and projected scenarios across Xizang Autonomous Region. Permutation importance diagnostics confirmed temperature sensitivity, and mean temperature of the coldest quarter (Bio11) demonstrates the highest model dependency.

**TABLE 2:** Relative contribution (%) and permutation importance of environmental variables to model prediction.

Variables	Descriptions	Percent contribution (%)	Permutation importance
Bio11	Mean temperature of the coldest quarter (°C)	48.5	88.3
HII	Human Impact Index, the human footprint	22.5	2.3
T_pH_H_2_O	Upper soil pH	21.5	0
Bio19	Precipitation of the coldest quarter (mm)	1.9	3.2
Awc_class	Available water storage (mm/m)	1.9	0
Elev	Elevation (m)	1.7	0.3
Bio18	Precipitation of the warmest quarter (mm)	1.6	5.7
Aspect	Aspect (°)	0.4	0

**FIGURE 4:**
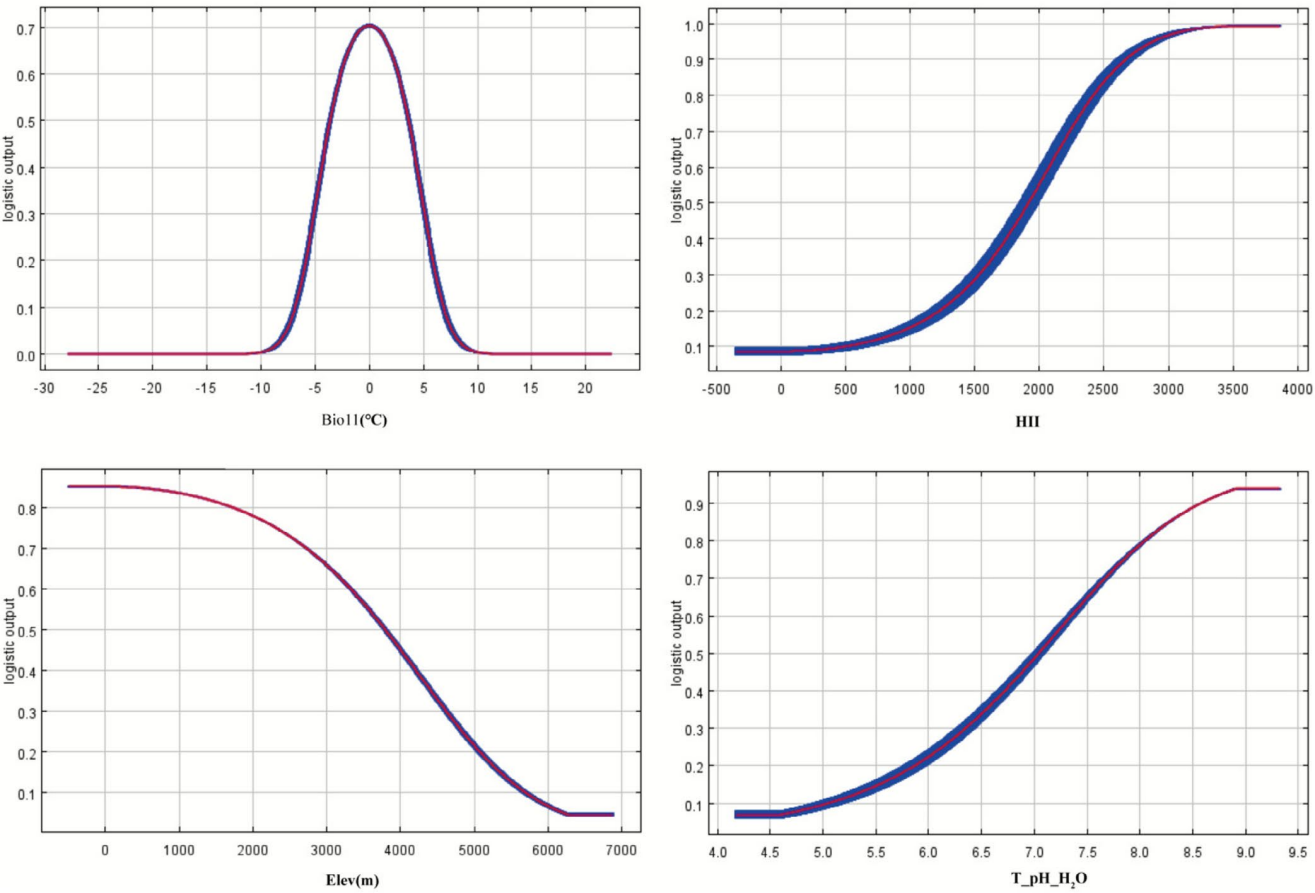
Response curves of four major environmental variables (biol1, HII, elev, T_pH_H2O). The red line is the mean value for the 10 MaxEnt runs, and the blue bar represents + standard deviation.

Jackknife analysis integrated with literature‐based variable selection identified four key environmental variables governing *C. bipinnatus* occurrence probability under isolated variable conditioning: mean temperature of the coldest quarter (Bio11), HII, upper soil pH (T_pH_H2O), and elevation (Figure 4). Response curve quantification delineated species‐specific environmental thresholds, with presence probability values between 0.5 and 1.0, indicating bioclimatic suitability ranges conducive to population establishment and range expansion. Ecophysiological analysis delineated species‐specific environmental optima for *C. bipinnatus* establishment, demonstrating thermal adaptation within −3.605°C to 3.590°C for mean coldest quarter temperature (Bio11), peaking at −0.033°C (P_max_ = 0.703). Anthropogenic facilitation thresholds revealed enhanced colonization likelihood above HII = 1917.24, reaching maximum probability (0.993) at 3519.62. Constraints of elevation showed a negative correlation above 3793.95 m, with optimal suitability at 85 m elevation (0.853 occurrence probability). Edaphic adaptation exhibited positive pH dependence beyond 7.044, achieving maximal fitness (*p* = 0.937) at upper soil pH = 8.906, collectively defining multidimensional niche boundaries.

### 3.2 Potential Risk Areas for *C. bipinnatus* Invasion Under Current and Future Climate Change Scenarios

Field investigations demonstrated that *C. bipinnatus* exhibits a distinct distribution pattern concentrated in the southeastern Xizang Autonomous Region, particularly within river valley ecosystems and anthropogenically disturbed areas. Geographically, its distribution extends from Gyirong County (Shigatse Prefecture) in the western limit to Markam County (Qamdo Prefecture) in the eastern boundary, with population concentrations strongly associated with human activity intensity across Lhasa, Shigatse, Shannan, Nyingchi, and Qamdo prefectures.

In Shannan Prefecture, established populations were documented in multiple urban centers including Gyaca, Sangri, Lhünzê, Lhozhag, Nêdong, Qonggyai, and Gonggar Counties. The Lhasa Municipality showed particularly dense aggregations within Chengguan District, Maizhokunggar, Damxung, Qüxü, Lhünzhub, and Dagzê Districts. Rapid expansion trends were observed in Shigatse Prefecture's Samzhubzê District, Yadong, Bainang, Dinggyê, Sa'gya, and Gyirong Counties. Verified occurrences in Qamdo Prefecture included Dêngqên, Baxoi, Karuo District, and Markam County, whereas Nyingchi Prefecture populations were confirmed in Bayi District, Mainling, and Gongbo'gyamda Counties. Notably, no specimens were detected in the northern plateau regions of Nagqu and Ngari Prefectures during systematic surveys.

**FIGURE 5:**
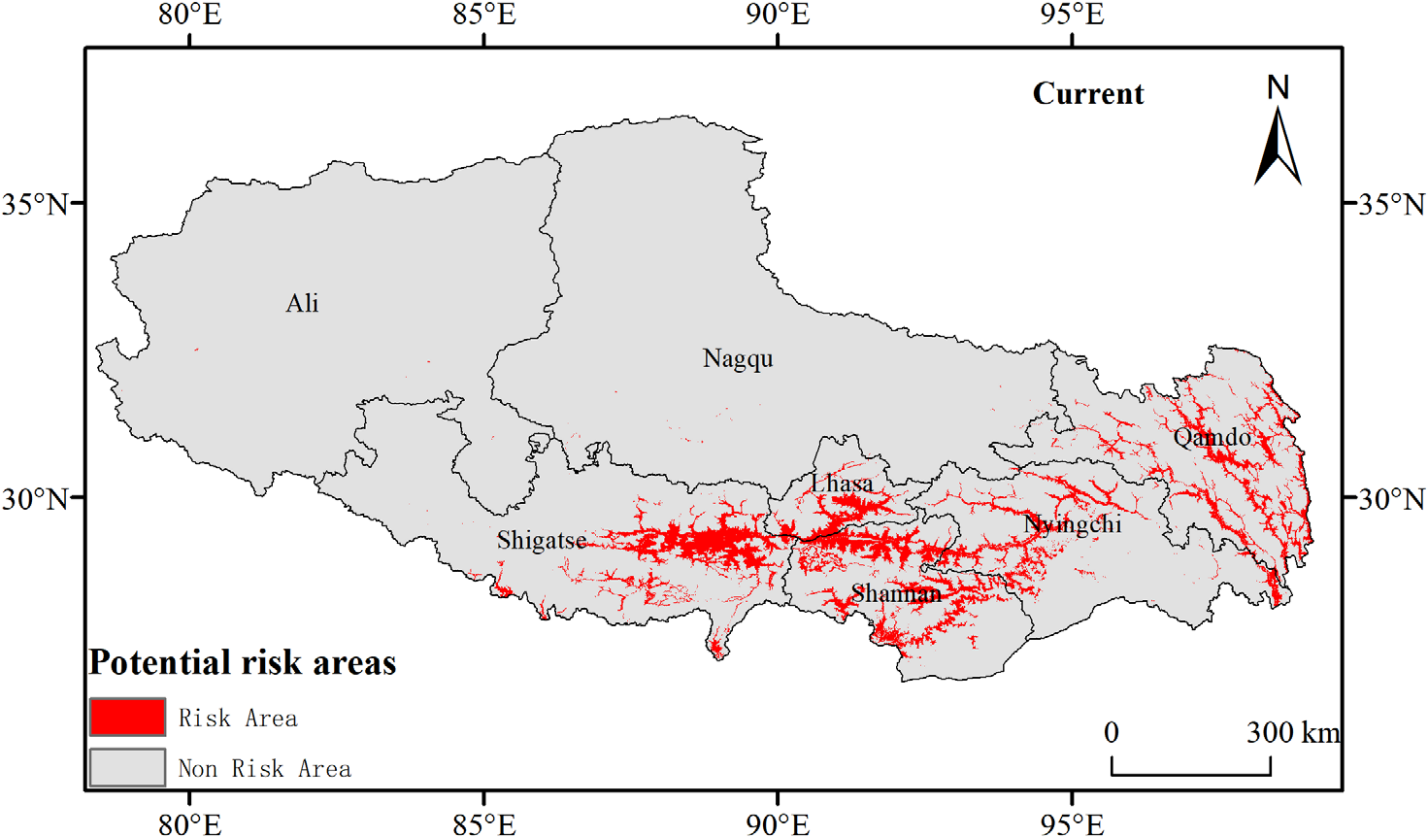
Predicted potential invasion risk areas of *C. bipinnatus* in Xizang using the MaxEnt model under the current climate (1970–2000).

The MaxEnt 3.4.4 model projections under contemporary climate scenarios demonstrated strong concordance with field survey data (Figure 5), revealing 6.87 × 10^4^ km^2^ of potential risk areas (5.59% of Xizang Autonomous Region's total land area). Spatial heterogeneity analysis showed high‐density clusters in Shannan and Lhasa Prefectures, with Shigatse Prefecture exhibiting marked expansion trends. Disjunct populations were documented in Nyingchi and Qamdo Prefectures, whereas sparse occurrences in northern plateau regions (Nagqu and Ngari Prefectures) were predominantly confined to river valleys and anthropogenic corridors, mirroring empirical field observation patterns.

Under future climate scenarios, *C. bipinnatus* exhibited divergent distribution patterns of potential risk areas, with projected contraction under the low greenhouse gas emission scenario (SSP1‐2.6) and progressive expansion under the carbon emission scenario (SSP5‐8.5) (Table 3).

**TABLE 3:** Proportion of risk areas of *C. bipinnatus* invasion in Xizang.

Climate change scenarios	Non‐risk area		Risk area	
	Area (×10^4^ km^2^)	Proportion (%)	Area (×10^4^ km^2^)	Proportion (%)
Current	115.98	94.41	6.87	5.59
SSP126‐2050	115.85	94.30	6.99	5.70
SSP126‐2090	116.90	95.16	5.95	4.84
SSP585‐2050	104.70	85.23	18.14	14.77
SSP585‐2090	100.44	81.76	22.41	18.24

**FIGURE 6:**
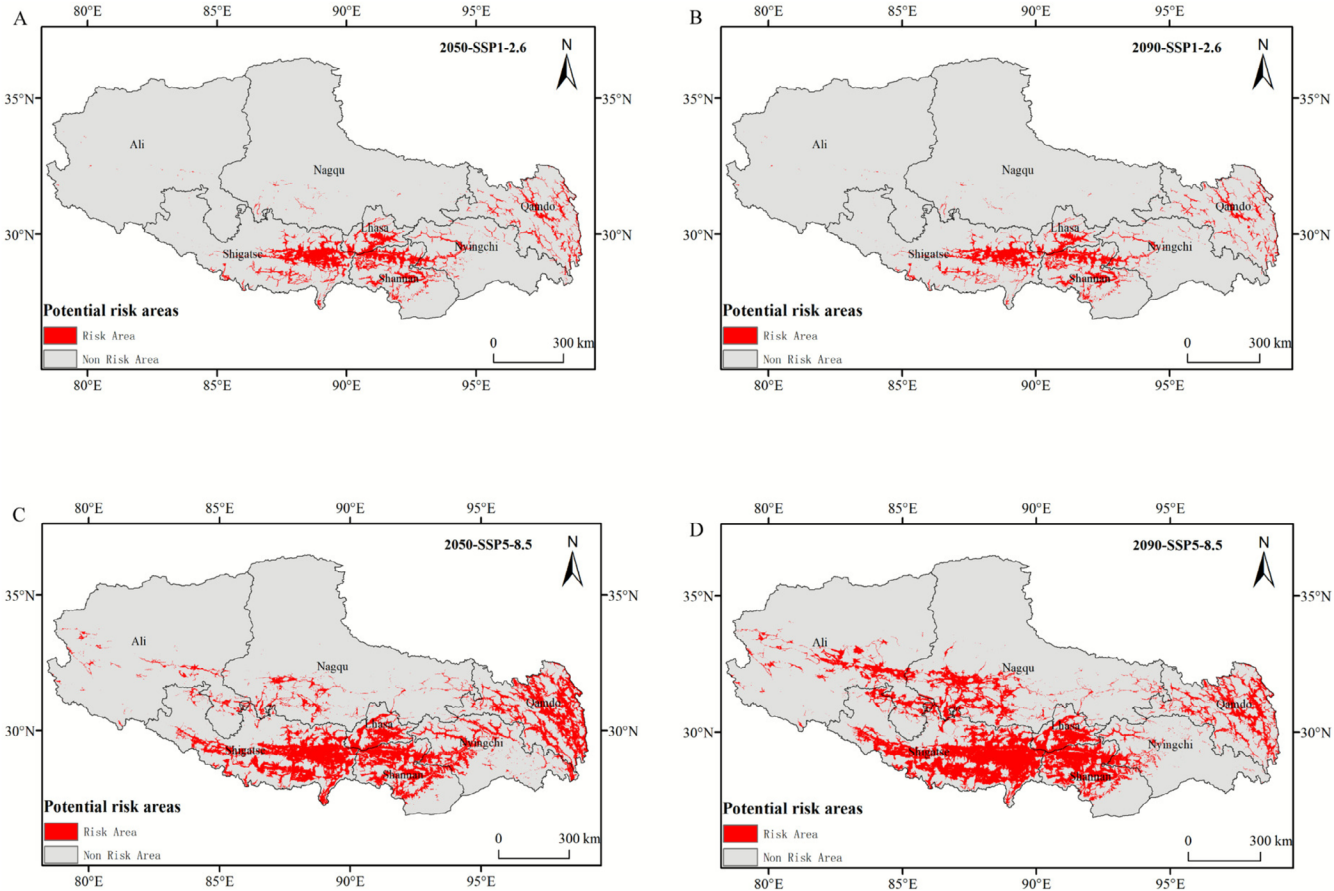
Predicted potential risk areas of *C. bipinnatus* invasion in Xizang using the MaxEnt model under the low greenhouse gas emission scenario (SSP1‐2.6) by (A) 2050 and (B) 2090. Predicted potential risk areas of *C. bipinnatus* invasion in Xizang using MaxEnt model under the high greenhouse gas emission scenario (SSP5‐8.5) by (C) 2050 and (D) 2090.

Under the SSP1‐2.6 scenario (Figure 6), the potential risk area for *C. bipinnatus* demonstrated phased dynamics, initially expanding to 6.99 × 10^4^ km^2^ (5.70% of Xizang's territory) by 2050 before subsequently declining to 5.95 × 10^4^ km^2^ (4.84%) by 2090 (Table 3), reflecting climate‐mediated range contraction mechanisms in a long‐term low greenhouse gas scenario.

Under the SSP5‐8.5 scenario (Figure 6), the potential risk area for *C. bipinnatus* is projected to expand from a baseline of 6.87 × 10^4^ km^2^ to 18.14 × 10^4^ km^2^ (14.77% of Xizang's land area) by 2050, with further progression to 22.41 × 10^4^ km^2^ (18.24%) by 2090 (Table 3). Comparative analysis across greenhouse gas emission scenarios revealed a consistent northwestward expansion trajectory.

### 3.3 Spatial Transfer Characteristics of Risk Areas for *C. bipinnatus*

Spatiotemporal analysis of *C. bipinnatus* habitat dynamics under two contrasting scenarios (SSP1‐2.6 and SSP5‐8.5) across mid‐century (2050) and end‐of‐century (2090) timelines revealed stable core habitats predominantly clustered in the Lhasa Municipality, Shigatse Prefecture, and Shannan Prefecture of Xizang Autonomous Region, with emerging northwestward expansion trajectories (Table 4).

**TABLE 4:** Changes in *C. bipinnatus* invasion risk areas from current to 2050 and 2050 to 2090 in Xizang under different climate change scenarios.

Climate change scenarios		Expansion (×10^4^ km^2^)	Stability (×10^4^ km^2^)	Contraction (×10^4^ km^2^)	Without change (×10^4^ km^2^)
SSP126	Current‐2050	1.23	5.39	1.10	115.13
	2050–2090	0.09	5.54	1.08	116.14
SSP585	Current‐2050	10.85	6.26	0.24	105.51
	2050–2090	6.80	14.27	2.84	98.94

**FIGURE 7:**
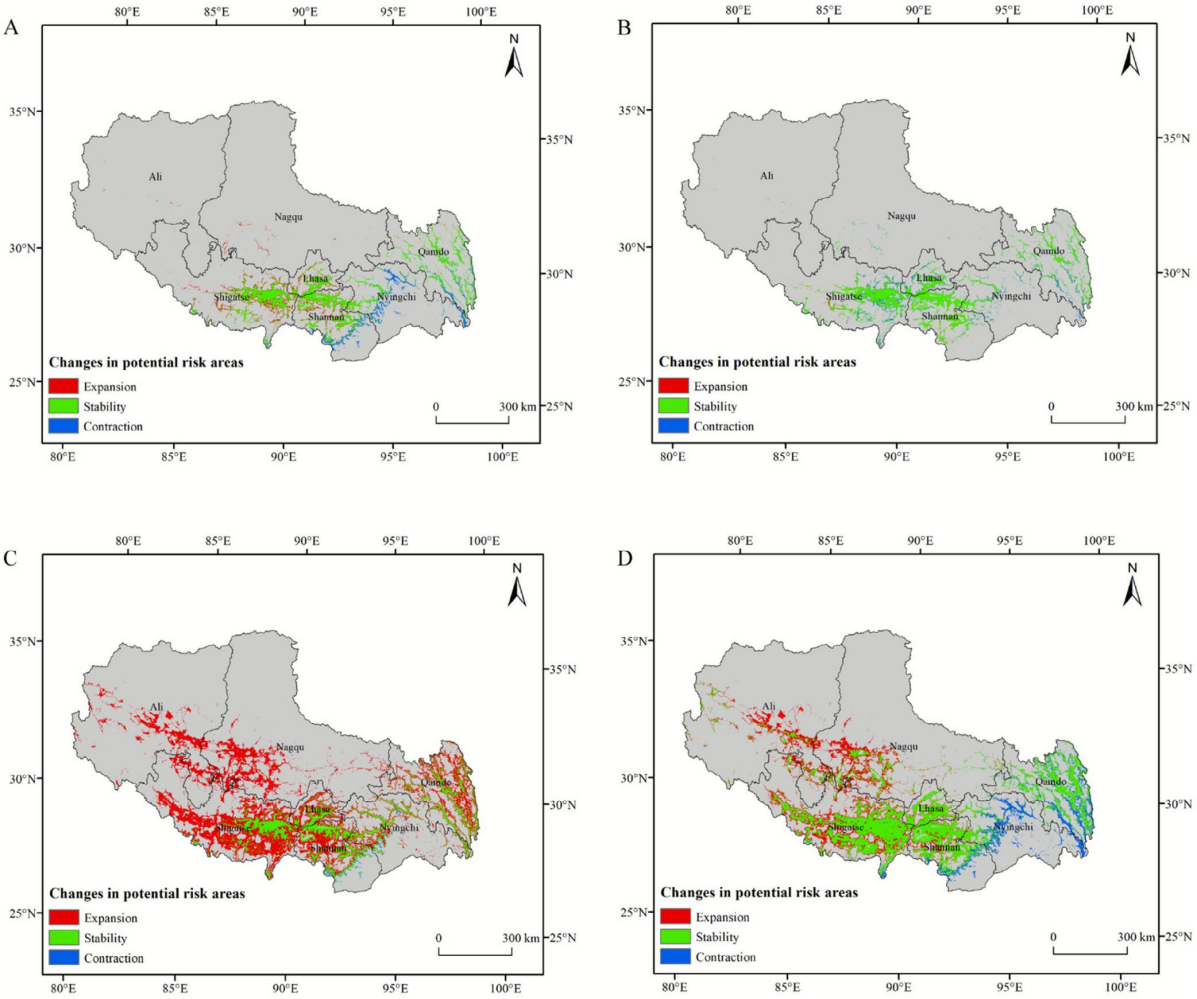
Spatial transfer characteristics of *C. bipinnatus* under the SSP1‐2.6 scenario for (A) current–2050 and (B) 2050–2090. Spatial transfer characteristics of *C. bipinnatus* under the SSP5‐8.5 scenario for (C) current–2050 and (D) 2050–2090.

Under SSP1‐2.6 scenario (Figure 7), by 2050, the ratio of expansion to contraction areas for *C. bipinnatus* potential risk zones is 1.23/1.1(Table 4), indicating limited expansion. Expansion occurs around Lhasa, Shigatse, and Shannan, whereas contraction is seen in southern Shannan, parts of Nyingchi, and some areas of Qamdo. By 2090, the expansion‐to‐contraction ratio shifts to 0.09/1.08, with expansion areas shrinking significantly. Expansion is minimal, only at the edges of the 2050 zones, and contraction extends further in southeastern Xizang. Most 2050 expansion areas become non‐risk zones, with only a few stable regions remaining. From the present to 2090, the overall expansion‐to‐contraction ratio is 1.32/2.18 (Table 4), showing an initial increase followed by a decrease in risk zones, with a general trend of contraction.

Under SSP5‐8.5 scenario (Figure 7), *C. bipinnatus* exhibited disproportionate range dynamics with expansion areas (10.85 × 10^4^ km^2^) substantially exceeding contraction zones (0.24 × 10^4^ km^2^) (Table 4). Geospatial analysis demonstrated pan‐prefecture expansion patterns across Xizang Autonomous Region: peripheral spread from existing risk zones in Qamdo, Shigatse, Shannan, and Lhasa Prefectures; novel colonization fronts in Nagqu and Ngari Prefectures; and fragmented contraction clusters concentrated in southern and southeastern regions, particularly within lower‐elevation valleys exhibiting intensified anthropogenic disturbance. By 2090, *C. bipinnatus* exhibited intensified range dynamics with 6.80 × 10^4^ km^2^ of additional expansion zones and 2.84 × 10^4^ km^2^ of emergent contraction areas (Table 4). The expansion front concentrated in western Lhasa Municipality and northern sectors of Shigatse, Nagqu, and Ngari Prefectures, whereas contraction nuclei clustered in southeastern Nyingchi and Qamdo Prefectures, demonstrating a distinct westward amplification and eastward attenuation pattern. Cumulative expansion from present‐day to 2090 reached 17.65 × 10^4^ km^2^ (14.37% of Xizang Autonomous Region). The distribution pattern of the potential risk area for *C. bipinnatus* is projected to shift from southeastern to northwestern Xizang.

**FIGURE 8:**
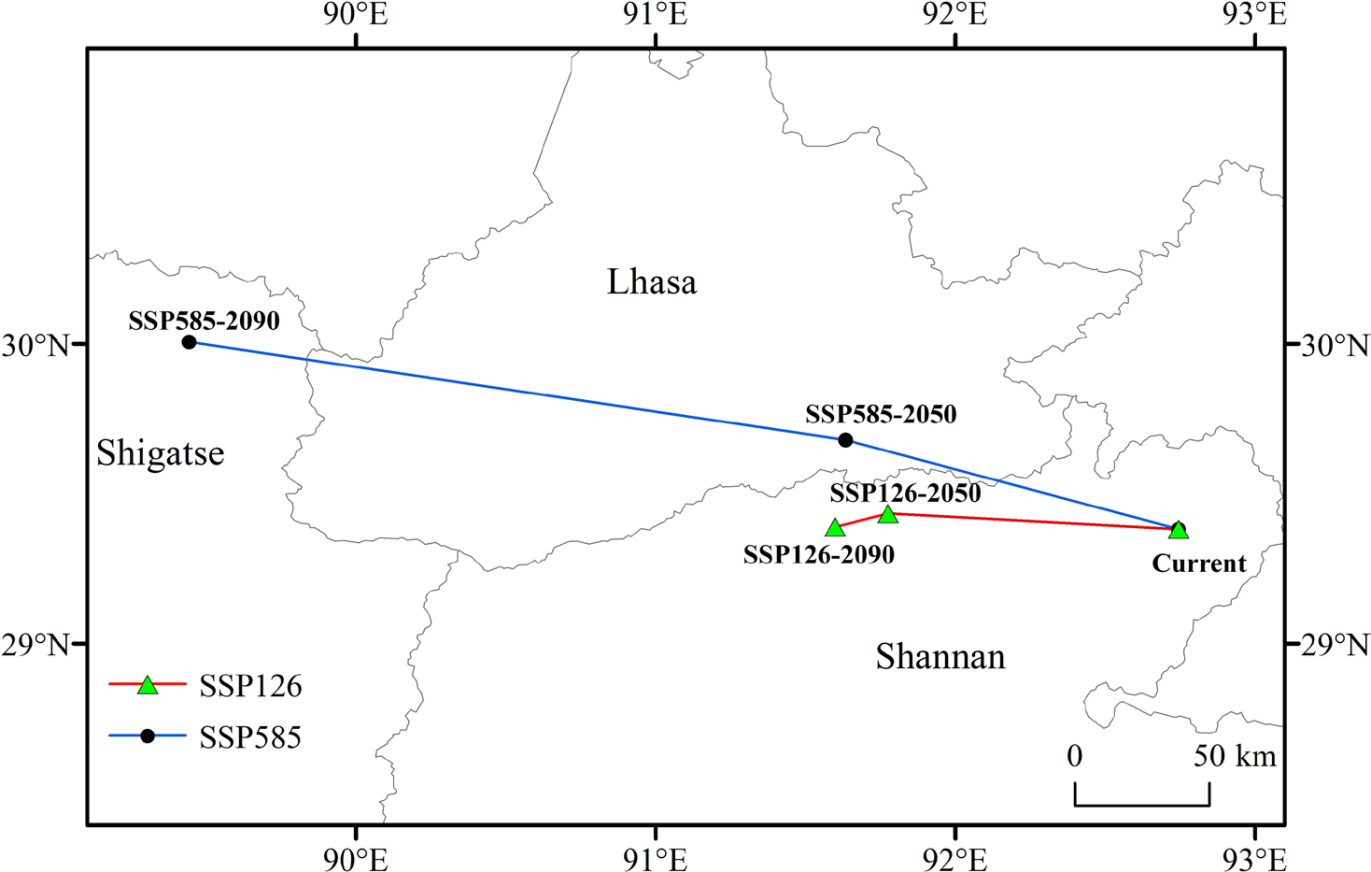
The centroid relocation trajectory of *C. bipinnatus* risk area in Xizang under different climate change scenarios.

Under the SSP1‐2.6 scenario, centroid trajectory analysis revealed dynamic shifts in *C. bipinnatus* distribution core areas (Figure 8). The contemporary centroid was geolocated at Gyaca County, Shannan Prefecture (92.74° E, 29.38° N). Projections indicated a compound migration vector: 107.73 km northwestward displacement to Nêdong District (91.77° E, 29.43° N) by 2050, followed by an incremental 19.66 km southwestward shift to Danang County (91.59° E, 29.39° N) by 2090, exhibiting a net northwestward trajectory (cumulative displacement: 127.39 km).

Under the SSP5‐8.5 scenario, centroid trajectory analysis exhibited sustained northwestward displacement vectors for *C. bipinnatus* distribution core areas, with an initial 123.5 km migration to Maizhokunggar County, Lhasa Municipality (91.63° E, 29.67° N) by 2050, followed by an additional 243.58 km displacement reaching Namling County, Shigatse Prefecture (89.44° E, 30.01° N) by 2090.

Comparative trajectory analysis under SSP1‐2.6 and SSP5‐8.5 scenarios demonstrated convergent northwestward displacement of *C. bipinnatus* distribution centroids, shifting from Gyaca County, Shannan Prefecture, to Chanang County, Shannan (SSP1‐2.6) and Namling County, Shigatse (SSP5‐8.5) with cumulative migration distances exceeding 100 km.

## 4 Discussion

Invasive alien species (IAS) often possess robust ecological adaptability and expansion capacity, enabling them to thrive in climates and regions where other plants struggle (Sun et al. 2023; Kumar Rai and Singh 2020). Species' geographic distributions are shaped by natural and human factors. However, it is important to note that data recorded in public databases may contain errors and not fully align with reality. Consequently, predictions of the geographic distribution of invasive species on the basis of such data may not accurately reflect their actual distribution patterns. The incorporation of field data, encompassing species presence/absence records and environmental variables, into species distribution models (e.g., Maxent models) has been demonstrated to enhance prediction accuracy. This is due to the fact that field surveys are designed to capture the environmental conditions and ecological niches occupied by target species. These models establish connections between species and their ecological requirements, identifying key environmental variables essential for species survival within specific geographic areas.

### 4.1 Effects of Environmental Variable Factors on the Distribution of *C. bipinnatus*

The findings of this simulation prediction of the potential risk area of *C. bipinnatus*, as determined by the Maxent model, indicated that the distribution of *C. bipinnatus* was predominantly constrained by three environmental variables: the average temperature of the coldest season (Bio11), the influence of human activities (HII), and the pH of the upper soil (T_pH_H2O). This suggests that the distribution of *C. bipinnatus* in Xizang is constrained by both climatic and soil conditions, whereas its distribution is more likely to be found in areas with high anthropogenic activities.

*C. bipinnatus* is a sun‐loving plant that thrives in poor soils but lacks cold tolerance (Olajuyigbe and Ashafa 2014). However, modeling results show an unexpected optimum average temperature of −0.033°C for the coldest season, which is unusually low for typical environments. When the model was restricted to current climate conditions, the optimal lowest quarterly mean temperature was 0.235°C. This discrepancy suggests that *C. bipinnatus* may have adapted to the unique low‐temperature, high‐altitude environment in Xizang through temperature‐adaptive evolution, similar to the cold‐tolerant gene expression observed in the invasive plant *Alternanthera philoxeroides* (Luo et al. 2020). Further investigation is needed to clarify the ecological and evolutionary mechanisms driving this adaptation. This study compares model outcomes under two greenhouse gas emission scenarios with field survey results, predicting that the optimal average temperature of the coldest quarter for *C. bipinnatus* will continue to decline, and its occurrence time will precede the June–August survey period. Amid global warming and wetting (He et al. 2023; Li et al. 2010), the potential risk area of *C. bipinnatus* is expected to extend to northern Xizang, where the average temperature of the coldest quarter is even lower.

Research indicates that biodiversity on the Qinghai‐Xizang plateau increases with soil pH (Shen et al. 2019; Wang et al. 2017), but the potential links of this to soil depth or the plants themselves need further study (Zhao et al. 2024). During environmental variable selection, it was found that when both upper—and lower—soil pH were considered, the contribution of lower—soil pH was much lower than that of upper—soil pH. This aligns with the characteristics of *C. bipinnatus* as an annual shallow—rooted herb, whose distribution is more affected by upper—soil pH. The environmental response curve shows that *C. bipinnatus* is more likely to occur in neutral and weakly alkaline soils, whereas acidic soils restrict its growth. This is consistent with the distribution patterns of other invasive Asteraceae plants (Gentili et al. 2018; Chai et al. 2012; Follmer et al. 2021; Ahmadi et al. 2024). Additionally, it has been pointed out that invasive plants can improve soil pH to create a favorable growth environment for themselves (Dar et al. 2023).

### 4.2 Features of Potential Risk Areas for *C. bipinnatus* Invasion Under Current Climate Change Scenarios

MaxEnt projections demonstrated strong congruence with empirical field surveys for *C. bipinnatus* risk zones. Current model outputs are mainly distributed in the urban areas and along the highways in the Xizang River Valley, showing a spatial structure of “centre aggregation + edge linear expansion”.

Notably, a spatial aggregation discrepancy emerged between model predictions and empirical data: field surveys revealed stronger clustering intensity in Shannan‐Lhasa transects compared to Shigatse, whereas model outputs depicted linear distributions in Shannan, contrasting with aggregated Shigatse clusters. This divergence may originate from (1) Geographic differences: similar to the Lhasa region, Shigatse is situated in a broad valley formed by the confluence of the Yarlung Tsangpo and Nyanchu Rivers (Szumańska et al. 2021; Nobis et al. 2018), which characterizes the spread of “leapfrog diffusion” that is dependent on human activities; (2) Ecological constraints in Shannan's Cona City and Lhünzê County sectors situated within southeastern biodiversity hotspots, where colonization is modulated by interspecific competition and niche differentiation processes, limiting cluster formation (Hu et al. 2015). Resolution of these biome‐specific distribution mechanisms warrants further investigation.

Through the results of the projected potential distribution areas of *C. bipinnatus* under contemporary climate scenarios, in summary, the expansion potential of *C. bipinnatus* will be in the following two paths: (1) The biodiversity hotspots of southeastern Xizangsuch as Shannan and Linzhi expand southeastward in a linear distribution belt, and eventually Shannan, Linzhi and Qamdo regions further show aggregated distributions; (2) the distribution of *C. bipinnatus* centered on the regions of Lhasa, Shigatse, and Shannan areas as the center to expand to the northwest, and eventually migrate to the northern areas of Xizang.

### 4.3 Spatial Transfer Characteristics of Risk Areas for *C. bipinnatus*

According to the Maxent prediction results, the center of mass of the potential risk area of *C. bipinnatus* migrates to the northwest under both greenhouse gas emission scenarios (SSP1‐2.6 and SSP5‐8.5) in the future, but the spatial distribution pattern of *C. bipinnatus* shows a significant difference.

Under the SSP1‐2.6 scenario, as climate change stabilizes, greenhouse gas concentrations decline, and human‐induced environmental impacts reduce over time, the potential risk area of *C. bipinnatus* shows a slight short‐term increase by 2050 but a long‐term decrease by 2090. Concurrently, the rate of spread of *C. bipinnatus* also exhibits a decline. This finding indicates that the expansion of *C. bipinnatus* may be somewhat constrained under this low greenhouse gas scenario.

Conversely, under the high greenhouse gas SSP5‐8.5 scenario, extreme climate changes, intensified global warming, and persistently high greenhouse gas concentrations mark human activities' aggravated environmental impact. In this region, the potential risk area for *C. bipinnatus* exhibits an annual expansion, reaching 22.41 × 104 km2 by 2090—representing 18.24% of Xizang's total area—with a sustained increase in its expansion rate. This finding underscores a substantially elevated invasion risk of *C. bipinnatus* under the high greenhouse gas emission pathway.

Research has shown that exotic plants undergoing prolonged adaptive evolution in invaded environments (Lambrinos 2001) may exhibit niche shifts, potentially occupying climatic niches distinct from their native ranges (Datta et al. 2019). This evolutionary mechanism introduces uncertainties in precisely predicting invasive species' distribution patterns (Guisan et al. 2014). Driven by escalating greenhouse gas emissions and global warming, *C. bipinnatus* likely developed progressive adaptations to the Xizang Plateau's extreme conditions over time. Projections indicate that by 2090, under the high greenhouse gas SSP5‐8.5 scenario, *C. bipinnatus* may spread quickly to northern Xizang with weak ecological barriers and low biodiversity. This could negatively impact nature reserves, such as the Qiangtang and Siling Co National Nature Reserves, in northern Xizang (Carneiro et al. 2024; Dayer et al. 2020). Therefore, emphasizing the prevention and monitoring of the invasive plant *C. bipinnatus* in the Xizang area is crucial.

This study sought to predict the invasion risk zone of the alien invasive plant *C. bipinnatus* under various climate change scenarios. The study was conducted at the Xizang scale, with the objective of providing scientific reference material for the prevention of local biological invasions and their management. However, the simulation results of *C. bipinnatus* in this study may not accurately reflect the distribution of risk zones for real *C. bipinnatus* , primarily because of the following reasons: (1) The current climate in this study was selected from the average of the real environmental climate data in the WorldClim dataset during the period of 1970–2000, which differs somewhat from the current climate. At the same time, the current climate in this study was averaged with the real environmental climate data from the WorldClim dataset, and there is some variability in the current climate. Concurrently, this study posits the hypothesis that the environmental variables (two soil factors, two topographic factors, and the human impact index) other than the climate environmental variables will remain constant in future climates. However, it does not take into account the dynamic effects of these factors that may change over time. (2) The anthropogenic human impact index (HII) considered in this study is only a simple weighting (human settlements, infrastructure, accessible areas, industrial areas, and nighttime lighted areas), whereas the actual specific human activity impacts are more complex and need to be combined with specific social, economic, and environmental composite variables.

Consequently, the contemporary prediction of invasive alien plants remains constrained, impeding the realization of a more realistic and comprehensive prediction and analysis of the invasion range and spatial change trend. However, in both contemporary and future scenarios, the distribution range of *C. bipinnatus* involves major cities in the Xizang Autonomous Region. Therefore, it is imperative to study its current invasion status and potential expansion areas. To effectively prevent and control the invasion of *C. bipinnatus* , a comprehensive management and monitoring system should be established, and scientific and reasonable preventive and control measures should be formulated by combining the needs of ecological protection and social development (Guan et al. 2023).

## 5 Conclusion

Under current climatic conditions, *C. bipinnatus* potential risk areas are distributed across central Xizang (Lhasa and Shigatse Prefectures), southeastern regions (Shannan and Nyingchi Prefectures), and eastern sectors (Qamdo Prefecture), with the contemporary distribution centroid located in Gyaca County, Shannan. Projections under contrasting SSP scenarios demonstrate convergent northwestward centroid migration toward northern plateau regions, yet reveal divergent spatial dynamics: the SSP1‐2.6 scenario induces 0.98 × 10^4^ km^2^ net contraction of potential risk areas, indicating climate‐mediated inhibitory effects on invasion spread, whereas the SSP5‐8.5 scenario drives 15.54 × 10^4^ km^2^ progressive expansion, highlighting exacerbated invasion risks under unrestricted greenhouse gas emission trajectories. The differences in greenhouse gas emission scenarios have caused a clear spatial reconfiguration of potential risk areas, significantly altering the biogeographic pattern of termites of Xizang.

The distribution of *C. bipinnatus* is mainly affected by the average temperature of the coldest quarter (Bio11), human activity impact (HII), upper soil pH (T_pH_H2O), and altitude. Among these, human activity has the most significant effect on the distribution of *C. bipinnatus* . Its influence begins at 1917.24 and increases with the index, eventually reaching 0.993, close to 1. It shows that increased human activity intensity greatly promotes the distribution and expansion of *C. bipinnatus* .

Comparative analysis of SSP1‐2.6 and SSP5‐8.5 scenarios reveals that high greenhouse gas emission trajectories significantly facilitate *C. bipinnatus* invasion across Xizang Autonomous Region under climate warming. The SSP5‐8.5 scenario drives substantial expansion of potential risk areas, exacerbating ecological threats to plateau ecosystems. To mitigate these bio‐security risks, the implementation of long‐term monitoring systems and integrated prevention frameworks becomes imperative. These measures constitute critical safeguards for maintaining Himalayan biodiversity and ensuring ecosystem stability amidst biological invasions.

## Author Contributions

**Xin Tan:** conceptualization (equal), data curation (equal), formal analysis (equal), investigation (equal), methodology (equal), resources (equal), validation (equal), visualization (equal), writing – original draft (equal), writing – review and editing (equal). **Zhefei Zeng:** methodology (equal), resources (equal), validation (equal), writing – review and editing (equal). **Wei Li:** methodology (equal), validation (equal), writing – review and editing (equal). **Jifeng Zhang:** methodology (equal), visualization (equal). **Norzin Tso:** investigation (equal). **Ngawang Norbu:** investigation (equal). **Mei Zhang:** investigation (equal). **La Qiong:** conceptualization (equal), methodology (equal), resources (equal), validation (equal), writing – review and editing (equal). **Junwei Wang:** conceptualization (equal), funding acquisition (equal), investigation (equal), methodology (equal), project administration (equal), resources (equal), supervision (equal), validation (equal), visualization (equal), writing – review and editing (equal).

## Funding

This study was funded by Investigation and Research on the Diversity of Invasive Alien Plants in Lhasa City, Natural Science Foundation of Xizang Autonomous Region, China (XZ202401ZR0028); Science and Technology Projects of Xizang Autonomous Region, China (XZ202402ZD0005); and Innovation Training Program for College Students of Xizang University (202510694035).

## Conflicts of Interest

The authors declare no conflicts of interest.

## Data Availability

Climate data can be obtained from Worldclim (http://www.worldclim.org), Human Influence Index from https://wcshumanfootprint.org/, and edaphic parameters from https://www.fao.org/soils‐portal/.
